# What Physicians Average per Year in New York

**Published:** 1898-02

**Authors:** 


PHYSICIANS are said to average only $1,500 per year in New York, and the reason may be found in these statistics: There are 114 dispensaries and 26 hospitals in New York County. In the 26 hospitals, in the year 1895, 75,368 patients were treated free, and in the dispensaries 661,803, making a total of 737,171. This is a goodly proportion out of a 1,851,000 population—nearly 40 percent. There have been 92,529 free visits of patients to hospitals, and 1,387,170 free visits of patients to dispensaries. In attendance upon the 114 dispensaries in 1895 were 949 medical men, which is 27 percent of all the physicians in the city, who number 3,430. Here we have more than 1,500,000 free visits and more than 1,000,000 free prescriptions, and more than one-quarter of all the physicians in the city of New York engaged in treating the population free of charge.—*The Engineer.*

